# Laboratory Investigation of Lignocellulosic Biomass as Performance Improver for Bituminous Materials

**DOI:** 10.3390/polym11081253

**Published:** 2019-07-29

**Authors:** Duanyi Wang, Zhiwei Cai, Zeyu Zhang, Xinquan Xu, Huayang Yu

**Affiliations:** 1 School of Civil Engineering and Transportation, South China University of Technology, Guangzhou 510000, China; 2 Institute of Highway Engineering, RWTH Aachen University, 52074 Aachen, Germany; 3 Guangdong Hualu Transport Technology Co. Ltd., Guangzhou 510000, China

**Keywords:** lignocellulosic biomass, bitumen modifier, workability, rheological tests, mechanical properties

## Abstract

Lignocellulosic biomass has gained increasing attention as a performance modifier for bituminous material due to the vast amount available, its low cost and its potential to improve the durability of pavement. However, a comprehensive study concerning both the binder and mixture performance of modified bituminous material with lignocellulose is still limited. This research aims to evaluate the feasibility of applying lignocellulose as bitumen modifier by rheological, chemical and mechanical tests. To this end, two lignocellulosic biomass modified bituminous binders and corresponding mixtures were prepared and tested. The chemical characterization revealed the interaction between lignocellulosic biomass and bitumen fractions. Rheological test results have shown that lignocellulosic modifiers improve the overall performance of bituminous binder at high, intermediate and low temperatures. The findings obtained by mixture mechanical tests were identical to the binder test results, proving the positive effect of lignocellulosic biomass on overall paving performance of bituminous materials. Although lignocellulosic modifier slightly deteriorates the bitumen workability, the modified bitumen still meets the viscosity requirements mentioned in Superpave specification. This paper suggests that lignocellulosic biomass is a promising modifier for bituminous materials with both engineering and economic merits. Future study will focus on field validation and life cycle assessment of bituminous pavement with lignocellulosic biomass.

## 1. Introduction

Lignocellulosic biomass is recognized as a renewable and abundant resource generated from numerous human activities. It is composed of lignin, hemicellulose and cellulose as major chemical constituents [1]. Among those, cellulose consists of different percentages of crystalline and amorphous structures. Both the physical and chemical properties of cellulose are closely related to the arrangement of the cellulose molecules [2]. Hemicellulose is a polysaccharides that composed of heterogeneous groups of pentose and hexose sugars [3]. Lignin is known as an amorphous heteropolymer with different phenylpropane units which are gathered together by different kind of linkages [4,5,6]. In recent decades, vast amount of lignocellulosic wastes has been generated around the world, from different sources including agriculture, construction, wood and furniture industries. In most cases this type of waste materials are either burnt or land filled, which results in environmental concerns like hazardous emission and land occupation. To date, only a small amount of lignocellulosic wastes has been utilized and recycled as useful bioproducts [7]. Due to its abundant quantity and high content of aromatic structures, lignocellulosic waste is a potential sustainable bio-resource which can be utilized as a modifier or alternative material for other industrially aromatic polymers.

Bitumen is a by-product generated by the refining process of the petroleum industry. In recent decades, bitumen has been extensively used as a binder to glue the loose aggregates for pavement construction [8]. Bituminous pavement, also known as flexible pavement, has been widely paved due to attractive advantages like improved smoothness, low traffic noise and easy maintenance. The performance of flexible pavement is closely related to the rheological properties of bituminous binder. To improve the durability of pavement, a series of bitumen modifiers have been developed and applied, including styrene butadiene styrene (SBS) [9,10], crumb rubber [11,12,13], bio oil [14], plastic [15] and different types of fibers [16]. The main components of raw bitumen are statures, aromatics, resins and asphaltenes, and the above-mentioned modifiers have good compatibility with the bitumen fractions. Especially, polymers like SBS have been recognized as the most effective modifiers to improve the overall performance of flexible pavement. Ascribed to advanced crude oil refining technology, the annual production of bitumen is decreasing, resulting in higher material cost for asphalt pavement construction [17]. As mentioned in previous research, certain chemical similarities exist between cellulose, lignin and bitumen, as they are both hydrocarbon materials composed mainly of carbon, hydrogen and oxygen [18]. Considering the abundant source and chemical structure, it is promising to utilize lignocellulosic biomass as a modifier or substitute for bituminous materials towards enhanced road performance. In addition, the application of lignocellulosic products as a partial substitute may alleviate the concern of petroleum-based bitumen production shortage.

There are a few recent studies about the application of lignocellulosic materials as bitumen modifier or alternative, and promising results have been obtained. Cellulose has been reported to be an effective bitumen modifier which enhances the mechanical performance of mixtures against both rutting and fatigue cracking [19,20]. Chen found that when blended with bituminous materials, lignin fiber (cellulose based commercial product) has good absorption to bitumen fractions [21]. Xu et al. studied the feasibility of lignin as partial alternative for bituminous binder by rheological evaluation. They found that lignocellulosic biomass brings stiffening effect in bitumen, improving the resistance to permanent deformation without deteriorating other properties [17]. In McCready and Williams′s study, lignin fiber was proven to be beneficial to the temperature sensitively of raw bitumen [22]. Pan found that lignin slows down the aging speed of bitumen [23]. Batista et al. proved that lignin modified asphalt binder exhibited superior performance in both rutting and cracking resistance. The incorporation of lignin also leaded to superior thermal stability [24]. Arafat et al. used three different types of lignin fibers for asphalt modification, and the positive effect in rutting, cracking, and moisture damage susceptibility brought by lignin was significant [25]. In the study of Xie and coauthors, the merits and defects of lignin as a sustainable bitumen modifier were illustrated in engineering and economic aspects [26].

Application of lignocellulosic materials as a bituminous modifier brings certain enhanced engineering performance and environmental benefits. The combination of biomass and bitumen may contribute to the development of both asphalt and paper industries as well as management of industrial waste. Although available studies have demonstrated the feasibility of utilizing lignocellulosic biomass as bitumen modifier, a comprehensive study concerning both the binder and mixture performance of bituminous material with lignocellulose is still limited. Hence, this study was conducted to obtain a clearer understanding of lignocellulose modified asphalt (LMA) binder and mixture by a series of experimental tests. To achieve this goal, rheological tests including Superpave performance grading, frequency sweep, multiple stress creep recovery test and liner amplitude sweep test were performed on LMA binders. In addition, corresponding mixture properties including Marshall stability, resistance to aging and moisture susceptibility were tested. It is expected that this paper can provide helpful information regarding the use of lignocellulose-based products as sustainable alternative for pavement materials.

## 2. Materials and Methods

### 2.1. Materials and Sample Preparation

#### 2.1.1. Materials

**Figure 1:**
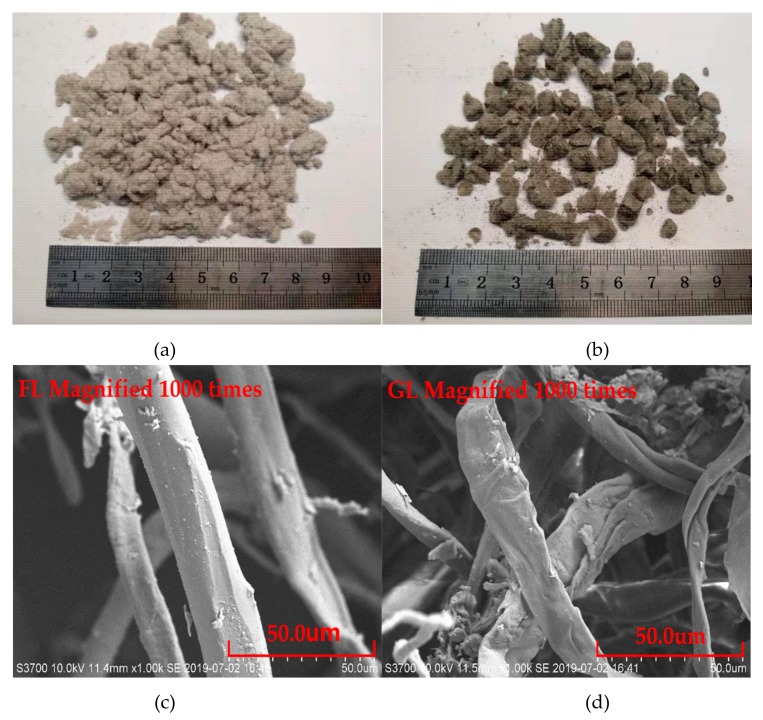
Lignocellulosic modifiers: (**a**) flocculent lignocellulose (FL) and (**b**) granulated lignocellulose (GL); (**c**) SEM of FL and (**d**) SEM of GL.

The Pen60/70 raw bitumen provided by Maoming Petrochemical Co. Ltd. (Guangdong, China) was blended with two types of lignocellulosic modifiers with different physical shapes (Figure 1). The lignocellulosic biomass modifiers were provided by Changzhou Lubisi New Material Technology Co. Ltd. (Changzhou, China) [27]. The flocculent lignocellulose (FL) was the conventional scattered lignocellulosic biomass (Figure 1a, c), while the granulated lignocellulose (GL) was prepared by condensing the scattered lignocellulose fibers by physical treatment (Figure 1b,d). The basic properties of lignocellulosic modifier are shown in Table 1. These two lignocellulosic modifiers, FL and GL, are organic fibers obtained by chemically treating natural hardwood, and then pulverized and extruded to produce granular and flocculent products. According to the information from manufacturer, the main components of the FL and GL were: 40–45 wt % cellulose, 15–20 wt % lignin (10–12 wt % insoluble Klason lignin and 6–10 wt % soluble lignin), 25–40 wt % hemicellulose and other compounds that appear in low percentages. Based on the scanning electron microscope (SEM) observation, it is noted that FL was more saturated compared to GL.

In this study, 10 mm gap-graded stone mastic asphalt (SMA10), which has been widely applied in the wearing course in South China, was chosen for bituminous mixture preparation. Table 2 presents the designed gradation information of aggregates. The diabase-type aggregates and fillers were provided by local asphalt plants. The aggregates were first washed and dried, and then sieved to meet the graduation requirement. Before being blended with hot bituminous binder, the sieved aggregates were placed in an oven at 20 °C above the blending temperature with bitumen for at least 4 h to remove moisture.

#### 2.1.2. Sample Preparation

The lignocellulose modified bituminous binders were prepared by wet process through blending FL and GL modifiers (4% by weight of raw asphalt) with raw bitumen using a high-shear radial flow impeller. A temperature of 160 °C and a speed of 4000 rpm were employed during the 60 min blending process. The material dosage and mixing condition were determined according to results of early trials [22]. The prepared bitumen with different lignocellulosic modifiers were labelled as GLA (GL modified asphalt) and FLA (FL modified asphalt) respectively.

For mixture preparation, Marshall design method was utilized to determine the optimum bitumen content. For mixtures with LMA binders and raw bitumen binder, the final asphalt contents were determined as 6.5% and 4.5% respectively. The blending temperature of bitumen and aggregates was set as 160 °C, identical to the preparing temperature of GLA and FLA. The compacting temperature was about 20 °C lower compared to the blending one. According to AASHTO and ASTM standards, specimens with 4% air voids were prepared for Marshall test [28] and indirect tensile stiffness modulus (ITSM) test. Samples with 7% air void were prepared for the indirect tensile strength (ITS) test [29]. 

### 2.2. Testing Program

#### 2.2.1. Penetration and Softening Point

Two empirical tests, namely, penetration and softening point tests, were performed in this study to characterize the empirical properties of test binders [30,31]. Penetration test evaluates the consistency of test bitumen. It determines the depth to which a needle (total weight 100 ± 0.1 g) can penetrate a bitumen sample under specified conditions of time (5 s) and temperature (25 °C). Softening point is regarded as an effective indicator for evaluating the high temperature performance of bitumen. In the softening point test, a steel ball with weight of 3.5 g is placed on the top surface of bituminous sample. The temperature of the bituminous sample is increased at a fixed rate. The temperature at which the steel ball falls is recorded as the softening point of bitumen. 

#### 2.2.2. Rotational Viscosity

The rotational viscosity of bituminous sample at the temperature of mixing and paving measured by rotational viscometer reveals the workability of bituminous samples. A Brookfield viscometer (AMETEK Brookfield Company, Middleboro, MA, USA) with 27# spindle was used, and the viscosity values at 135 °C (Superpave PG binder characterizing temperature) and 160 °C (the preparing temperature of modified binders) were measured [32].

#### 2.2.3. Performance grade (PG) in Superpave Specification

The performance grading of test binders was characterized by the dynamic shear rheometer (DSR, Malvern Kinexus Lab+, Malvern analytical Company, UK) test and bending beam rheometer (BBR, CANNON Instrument Company, State College, PA, USA) test [33]. The collected Superpave rutting parameter and fatigue parameter were employed to characterize the high and intermediate temperature performances of test binders, respectively. Unaged and short-term aged binders were used for Superpave rutting parameter measurement (with 25 mm-diameter plates) and long-term aged binders were used for Superpave fatigue parameter measurement (with 8 mm-diameter plates). The Superpave rutting factor test increased from 64 °C with an interval of 6 °C until the measured rutting factor was smaller than the threshold value from AASHTO M320 [34], i.e., 1.0 kPa for unaged binder and 2.2 kPa for short-term aged binder. Conversely, the initial test temperature of fatigue factor tests was set as 25 °C. The test temperature decreased with a decrement of 3 °C until the fatigue factor was larger than 5000 kPa. The BBR tests were conducted to evaluate the low-temperature performance, i.e., low temperature thermal cracking performance, of the binders according to AASHTO T313. Long-term aged samples were tested in a temperature fluid bath with a constant load (980 ± 50 mN) for 240 s. The BBR tests were started at −6 °C with a decrement of 6 °C. The critical parameters obtained from the BBR tests included the creep stiffness and m-value. 

#### 2.2.4. Other Viscoelastic Properties

Multiple stress creep recovery test was conducted to further characterize the permanent deformation resistance of test binder using the DSR at 60 °C [35]. The samples were subjected to creep-recovery cycles. Each creep-recovery cycle consists of a 1 s-creep stress followed by 9 s of recovery. Ten creep and recovery cycles were performed at a stress level of 0.1 kPa followed by another ten cycles at a stress level of 3.2 kPa. Three parameters including the average percent recovery (*R*%), non-recoverable creep compliance (*J*_nr_), and stress sensitivity parameter (Jnr-diff) were recorded to evaluate the high temperature performance of test binders.

The fatigue behavior of long-term aged test binders under various strain levels were evaluated by the liner amplitude sweep (LAS) test in accordance with AASHTO TP101-14 [36]. The LAS test consisted of two parts: frequency sweep and linear amplitude sweep. Frequency sweep was conducted by using the DSR at intermediate pavement temperatures at a strain level of 0.1% over frequencies ranging from 0.2 to 30 Hz. Once the frequency sweep test was completed, the linear amplitude sweep was performed on the test binder at a frequency of 10 Hz over a strain range of 0–30%. The number of cycles to failure at strain levels of 2.5% and 5% were calculated using the viscoelastic continuum damage (VECD) method.

In addition to the above-mentioned tests, the frequency sweep was carried out on GLA and FLA binders to characterize their overall rheological properties. Master curves (reference temperature at 60 °C), based on the time–temperature superposition principle, were obtained from frequency sweeps (The test was conducted from 76 to 4 °C, with a 12 °C reduction in temperature over the frequency range of 0.01 to 30 Hz).

#### 2.2.5. Molecular Weight Distribution

To reveal the interaction between lignocellulose and asphalt fractions, the molecular weight distribution of test binders was evaluated by gel permeation chromatography (GPC) test. A P230 Elite GPC was used to separate the constituents of the asphalt binders based on molecular size. Each sample was dissolved into Tetrahydrofuran (THF) and then filtered through a 0.2 µm Polytetrafluoroethylene (PTFE) syringe filter prior to being placed into the injection module. A polystyrene solution (1 mg/mL, the *M*_w_ of polystyrene is known) was used for column calibration. The chromatographic columns (PLgel 3 lm Mixed-3 + PLgel 5 lm 103 Å) were used in the GPC tests. During the GPC test, the bitumen-THF solution was drained through columns and allowed to flow at a rate of 0.5 mL/min, and the temperature of the columns were maintained at 40 °C. The components′ concentrations in the eluent were recorded using a differential refractometer, and the resulting chromatogram was analyzed to obtain the molecular size distribution.

It should be noted that only part of the GLA and FLA samples could be dissolved in the THF solvent. The solubility of GLA and FLA in THF was 98.9% and 98.6%, respectively. The solubility of GL and FL in Pen 60/70 was measured as 72.3% and 63.9%. However, the solubility of GL and FL in THF was only 11.8% and 11.2%, respectively, therefore, GPC tests were not performed on the original lignocelluloses.

#### 2.2.6. Fourier-Transform Infrared Spectroscopy (FTIR) 

Fourier-transform infrared spectroscopy (FTIR) was used to characterize the functional groups and chemical bonds of the test samples. The instrument used in the test was Bruker Vertex 70 (Billerica, MA, USA). The test was performed by pressing a test sample to pellets (about 1 mm thick) and then scanning by the infrared to obtain the infrared spectroscopy ranging from 4000 to 500 cm^−1^. 

#### 2.2.7. Marshall Stability and Flow Value

The standard Marshall test were conducted to characterize the deformation resistance of asphalt mixtures according to ASTM D6927-15 [28]. Marshall stability and flow value of asphalt mixtures were collected. Marshall stability refers to the maximum force the samples can withstand at the standard loading condition, namely, a constant loading speed of 50 mm/min. The amount of deformation of the samples when loaded to failure is expressed as the flow value. Before Marshall test, samples were placed into a 60 °C water bath for 30 min or 48 h (to evaluate the resistance to water damage).

#### 2.2.8. Moisture Susceptibility

The moisture susceptibility was quantified by the ratio of the indirect tensile strength after and before moisture conditioning of asphalt mixture sample. 25 °C was set as the testing temperature of ITS [29]. A vertical loading with a loading rate of 51 mm/min is applied through the dimeter, until the samples are split. For each type of asphalt mixture, six specimens were prepared and be divided to two groups, i.e. the control group and the freeze–thaw group. ITS tests were conducted on the control group before any conditioning. The freeze–thaw group were saturated by vacuum pump with water, and then subjected to one freeze–thaw cycle, i.e., a freeze phase at −18 °C for 16 h followed by a thaw phase at 60 °C for 24 h. The freeze–thaw specimens were placed in a 25 °C water bath for 2 h before the ITS test.

#### 2.2.9. Indirect Tensile Stiffness Modulus

Indirect tensile stiffness modulus (ITSM) tests were employed for investigating the stiffness of asphalt mixture [37]. The ITSM of both original and long-term aged asphalt mixture were collected at 20 and 30 °C. The aged mixtures were prepared by placing compacted mixtures in an 85 °C oven for five days, which is considered to be able to simulate the effect of long-term (5–10 years) aging that might occur on a pavement after construction. The indirect tensile stiffness modulus ratio (ITSMR), which equals to the ratio of the ITSM after and before the long-term aging, was calculated as an indicator of the samples′ aging sensitivity.

## 3. Results and Discussion 

### 3.1. Rheological Tests

#### 3.1.1. Empirical Parameters

**Figure 2:**
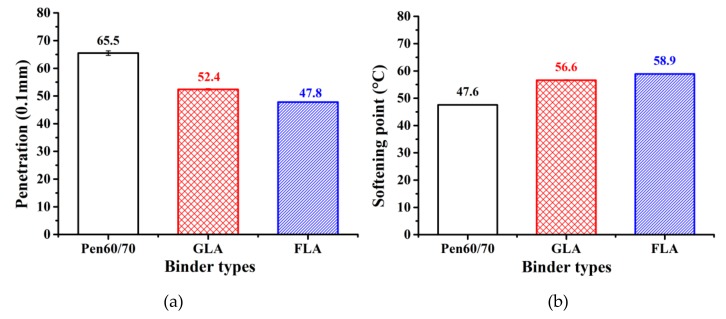
Bitumen empirical test results: (**a**) penetration and (**b**) softening point.

Penetration, ductility and softening point are known as the three empirical parameters of bituminous binder. Based on the preliminary test results, ductility didn′t seem to be a reliable parameter for LMA binders, since the heterogeneity caused by lignocellulosic modifiers might result in premature breakage, preventing the test to proceed normally. Therefore, only penetration and softening point tests were performed, and the results are shown in Figure 2. It is observed that both GL and FL decreased the penetration value of Pen60/70, making the binder thicker. Compared to Pen60/70, the softening point values of GLA and FLA were 9.0 and 11.3 °C higher, respectively. Those results indicated that the LMA binders should exhibit superior performance in high service temperature. By comparison, the effect of FL on the empirical parameters was more significant than that of GL. 

#### 3.1.2. Workability

**Figure 3:**
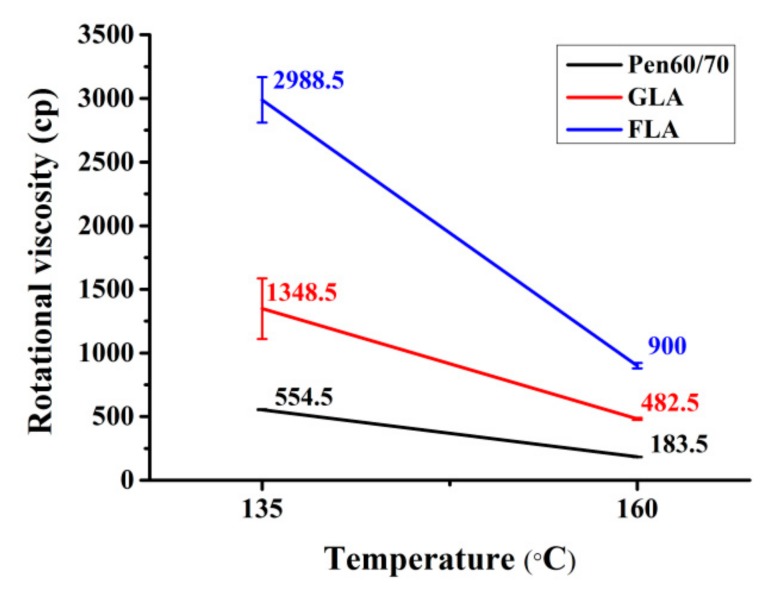
Rotational viscosity test results.

Rotational viscosity of bitumen has been a widely used parameter to determinate the appropriate blending temperature of bituminous mixtures. The viscosity values of test binders at 135 and 160 °C are shown in Figure 3. Be consistent with previous studies, the incorporation of lignocellulosic modifiers resulted in higher viscosity, indicating poorer workability [17,22]. This is ascribed to the stiffening effect of lignocellulosic fibers, making the bituminous binder thicker and more viscous. The negative effect on workability brought by FL was more pronounced. Based on the requirement of AASHTO specification, bitumen viscosity should be less than 3000 cp to ensure satisfactory compactable nature of the mixture [32]. It is noted that both FLA and GLA meet this requirement at 135 °C, suggesting acceptable workability of LMA binders. 

#### 3.1.3. Rutting Resistance

**Figure 4:**
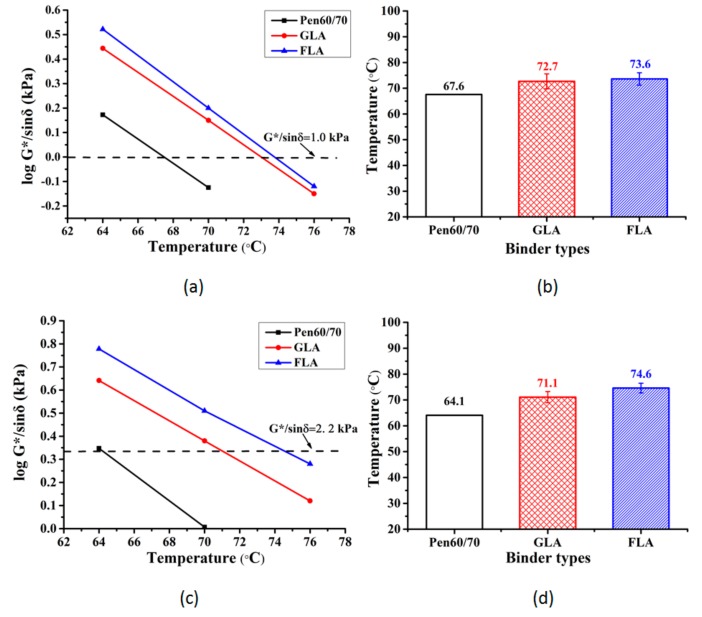
Superpave rutting factor test results: (**a**) rutting factor (unaged); (**b**) failure temperature (unaged); (**c**) rutting factor (short-term aged) and (**d**) failure temperature (short-term aged).

The binder performance at high temperature were evaluated by two tests, i.e., the Superpave rutting factor (G*/sinδ) test and MSCR test. Figure 4a and 4c shows the relationship between G*/sinδ and testing. Figure 4b and 4d illustrates the test results of failure temperature, which corresponding to the specific temperature when G*/sinδ is 1.0 kPa for unaged bitumen and 2.2 kPa for short-term aged bitumen. Based on Figure 4, lignocellulosic modifiers increased the failure temperature of Pen60/70 in both unaged and short-term aged stages. According to AASHTO specification, Pen60/70 met the requirement of PG64 while GLA and FLA can be labeled as PG70. By comparison, the difference between GLA and FLA was not obvious.

Table 3 shows the non-recoverable creep compliance (*J*_nr_) and percentage of recovery (*R*%) obtained by MSCR test. As Table 3 shows, the raw bitumen exhibited the highest *J*_nr_ value at both 0.1 and 3.2 kPa stress level. By comparison, GLA and FLA had smaller *J*_nr_ values, suggesting enhanced rutting resistance, which is consistent with the results obtained by both Superpave G*/sinδ test and softening point test. The *R*% results shown that bitumen with lignocellulosic modifiers recovered more than raw bitumen during the recovery period. The higher recovery rates of LMA binders have proven that the incorporation of lignocellulose results in more elastic behavior of bituminous binder, which allows more deformations to recover.

#### 3.1.4. Fatigue Resistance

**Figure 5:**
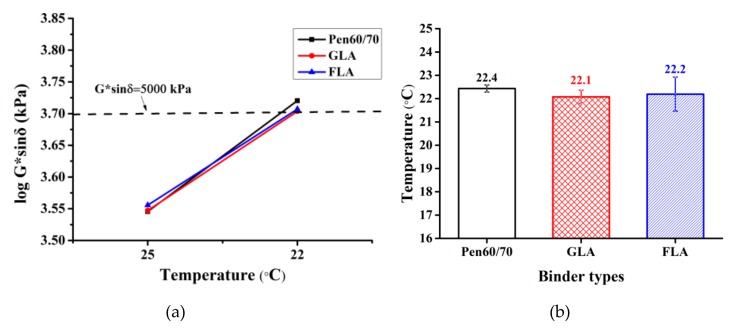
Superpave fatigue factor test results: (**a**) fatigue factor (long-term aged) and (**b**) failure temperatures (long-term aged).

The binder performance under repeated loading was characterized by both Superpave fatigue factor (G*sinδ) test and LAS test. The relationship between G*sinδ and testing temperature is shown in Figure 5a. Figure 5b presents the fatigue failure temperatures, which corresponding to the critical temperature that the G*sinδ value of test binder is equal to 5 MPa. As expected, the G*sinδ values of all specimens raised with declining test temperature. Figure 5b indicated that the difference among fatigue failure temperatures of three test binders were not obvious. The results shown that effect of lignocellulosic modifiers on fatigue resistance of bitumen is limited. 

**Figure 6:**
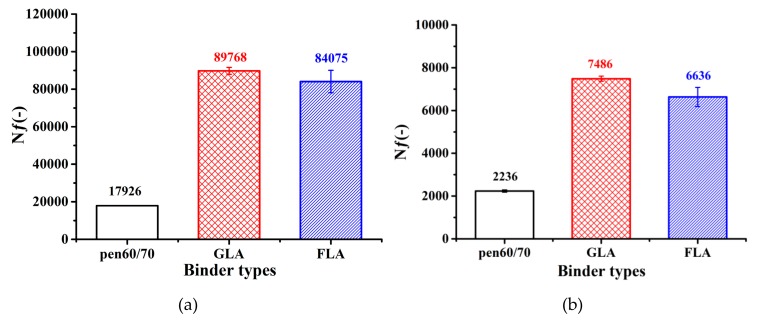
Liner amplitude sweep (LAS) test results: (**a**) applied strain of 2.5% and (**b**) applied strain of 5.0%.

LAS test evaluates the fatigue performance of bituminous binder in an accelerated way. Figure 6 shows the fatigue lives of test samples at 2.5% and 5% applied strains. Higher cycles to fatigue (*N*_ƒ_) refer to superior fatigue resistance. The fatigue lives of LMA binders are 4–5 times of those of Pen60/70 binder at both applied strain levels, indicating that the incorporation of lignocellulose modifiers brings extension of fatigue life. It is also observed that the Superpave fatigue factor test and LAS provided inconsistent fatigue performance prediction for LMA binders. Early publications have proven that the Superpave fatigue factor is not a reliable fatigue index for modified bitumen, as it is a stiffness-based parameter without considering the accumulated damage of test sample [38,39]. Therefore, according to the LAS evaluation, it is believed that lignocellulosic modifiers are beneficial to the fatigue resistance of raw bitumen.

#### 3.1.5. Low Temperature Performance

The anti-cracking performance at freezing temperature was evaluated by BBR test. The creep stiffness and creep rate values of test binders at −6, −12 and −18 °C were recorded, as Table 4 shows. Bitumen with lower stiffness and greater m-value has superior resistance to thermal cracking and better capability to sustain thermal stresses. By comparison, the LMA binders exhibited lower creep stiffness and higher m-value than raw bitumen. At −6 °C, all binders met the requirements of stiffness (<300 MPa) and m-value (>0.3) by AASHTO T313 [33]. However, Pen60/70 failed to pass the requirement at −12 °C, while GLA and FLA passed. Therefore, the LMA binders are believed to be applicable in cold regions.

#### 3.1.6. Overall Rheological Behavior

As mentioned in Section 2.2.4, the frequency sweep was performed on test binders at different temperatures and frequencies. Based on the frequency-temperature superposition principle, the master curves of complex modulus (G*) at 60 °C were developed by the following procedures: Firstly, the Williams–Landel–Ferry (WLF) formula (Equation (1) and Equation (2)) was substituted into the sigmoidal function (Equation (3) and yields Equation (4)); Secondly, nonlinear surface fitting was performed with Equation (4), obtaining parameters *C_1_* and *C_2_*. Finally, the parameters *C_1_*, *C_2_* and WLF equations are used to get the best fit and obtain the single master curve. The parameters of the sigmoidal function and WLF formula are presented in Table 5.

(1) $$log(a(T))=\frac{-C_{1}\Delta T}{C_{2}+\Delta T}$$

where a(T) is the shifting factor at specific temperature *T*, *C*_1_ and *C_2_* are model constants, and △*T* is the temperature difference between the test temperature and the specified temperature.

(2) log(ξ)=log(f)+log(a(T))

where ξ and ƒ refer to the reduced frequency at the specified temperature and the frequency at the test temperature, respectively.

(3) $$\log(G^{*})=\delta +\frac{\alpha }{1+e^{\beta +\gamma \log(\xi )}}$$

where β, γ are the shape parameters of the equation, α and δ is the span of G* values and the minimum modulus value respectively.

(4) $$\log(G^{*})=\delta +\frac{\alpha }{1+e^{\beta +\gamma (log(f)+\frac{-C_{1}\Delta T}{C_{2}+\Delta T})}}$$

**Figure 7:**
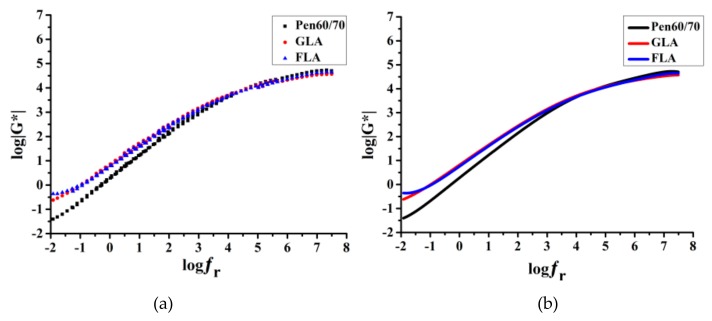
Master curves of test binders: (**a**) scatters of test results and (**b**) sigmoidal fitting curves.

Figure 7 shows the obtained master curves of test binders. Figure 7a is the scatters of test results and Figure 7b is the sigmoidal fitting curves. According to the time–temperature superposition principle of viscoelastic materials, low frequency corresponds to high temperature and vice versa. It is also noted that the bitumen modified by lignocellulose have obviously higher modulus than Pen60/70 at low frequencies, indicating superior rutting resistance. At high frequencies, Pen60/70 had the greatest modulus, followed by FLA and GLA. The obtained findings based on master curves were in accordance with results of MSCR and BBR tests.

### 3.2. Chemical Tests 

#### 3.2.1. Molecular Weight Distribution

**Figure 8:**
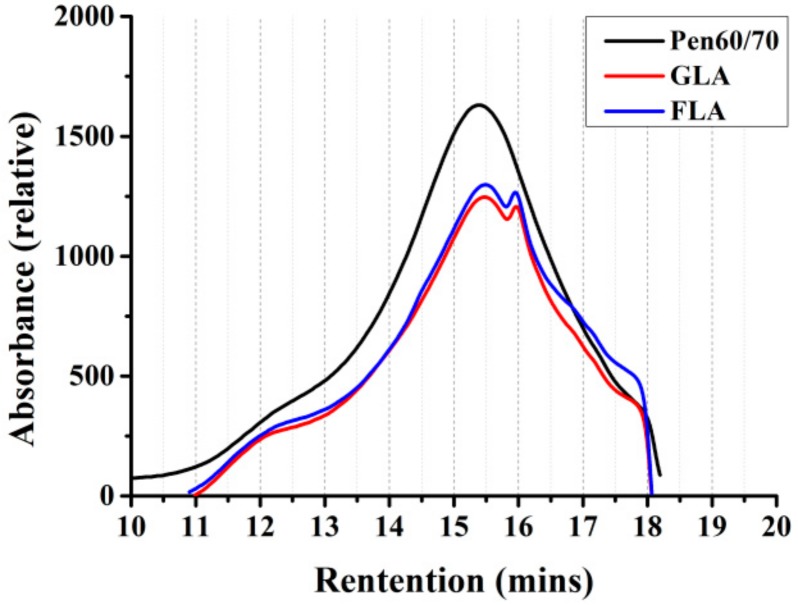
Gel permeation chromatography (GPC) chromatograms of test binders.

Figure 8 exhibits the chromatograms obtained by GPC test of the bituminous binders. According to previous studies about GPC characterization, the constituents of bitumen can be categorized as several groups by molecular weight (*M*_w_) [14,40]. In this research, the collected chromatograms are mainly based on retention time from 10 to 18 min, the corresponding *M*_w_ range is from 4,8386 to 125. Obviously, main peaks of all binders are mainly at 15.5 min, and the GLA and FLA binders have an additional small peak, at around 16 min. 

**Figure 9:**
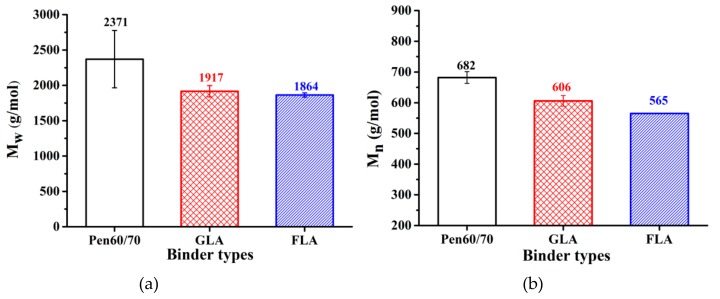
GPC result analysis: (**a**) weight-average molecular weight and (**b**) number-average molecular weight.

Table 6 shows the results of GPC test based on numerical statistics analysis. Five parameters were selected to illustrate the molecular weight distribution of test binders, including the peak molecular weight (*M*_p_), number-average molecular weight (*M*_n_), weight-average molecular weight (*M*_w_), z-average molecular weight (*M*_z_) and dispersity (Đ = *M*_w_/*M*_n_). Figure 9a,b show the weight-average molecular weight and number-average molecular weight, it is obvious that the molecular weight of GLA and FLA is much lower than that of raw bitumen. This could be ascribed to the insolubility of the fraction of the samples. The reaction products between lignocellulosic fiber and virgin binder may not dissolve in THF which decreases the molecular weight of asphalt binder. In addition, the molecular weight of lignocellulosic fibers dissolved in THF may lower than that of the virgin binder which also could be the reason for the decrease of modified binders’ molecular weight.

#### 3.2.2. Fourier-Transform Infrared Spectroscopy

**Figure 10:**
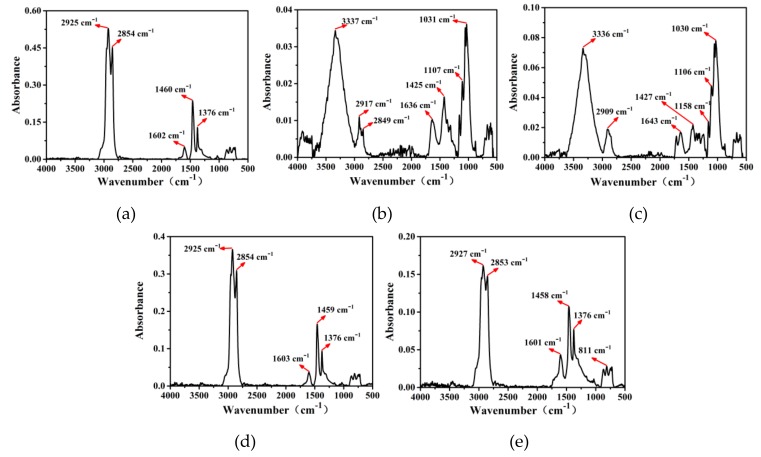
Fourier-transform infrared spectroscopy (FTIR) test results: (**a**) raw bitumen; (**b**) GL; (**c**) FL; (**d**) GLA and (**e**) FLA.

Figure 10a–c shows the FTIR test result of raw bitumen, GL and FL, respectively. In Figure 10a, the major bands around 2925 cm^−1^ are caused by the C–H stretching vibration of the alkyl group, and the peak of 2854 cm^−1^ is the stretching vibration of the aliphatic C–H. As shown in Figure 10b,c, for both GL and FL, the main absorbance peak appeared at the wavenumber of 3337 and 1031 cm^−1^. The peaks around 3337 cm^−1^ are associated with the N–H or O–H stretching vibration of the amino groups and hydrogen-bonded hydroxyl groups, while that around 1031 cm^−1^ may be due to the stretching of C–O and S=O. 

Figure 10d, e presents the FTIR spectra of GLA and FLA, it can be clearly seen that the functional groups of GLA and FLA are similar to virgin bitumen but differ in peak area. Compared to the FTIR spectra of GL and FL, the peaks of GLA and FLA near 3337 cm^−1^ are not significant, which may be caused by a chemical reaction between functional groups. Obviously, the GLA and FLA have similar majority bands, but the peak areas differ greatly. This phenomenon may be due to the difference in the degree of dissolution and reaction of GL and FL in bitumen fractions.

### 3.3. Mixture Test

#### 3.3.1. Marshall Stability and Flow Value

**Figure 11:**
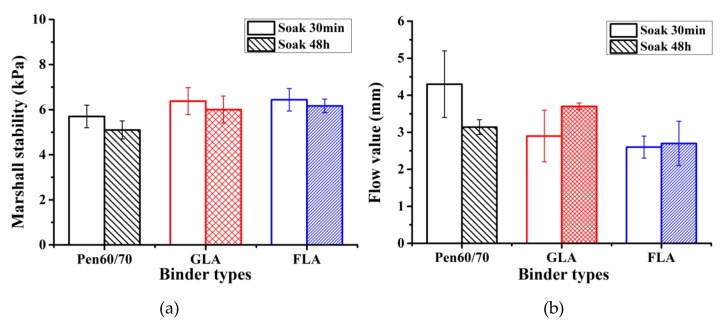
Marshall test results: (**a**) Marshall stability and (**b**) flow value.

**Figure 12:**
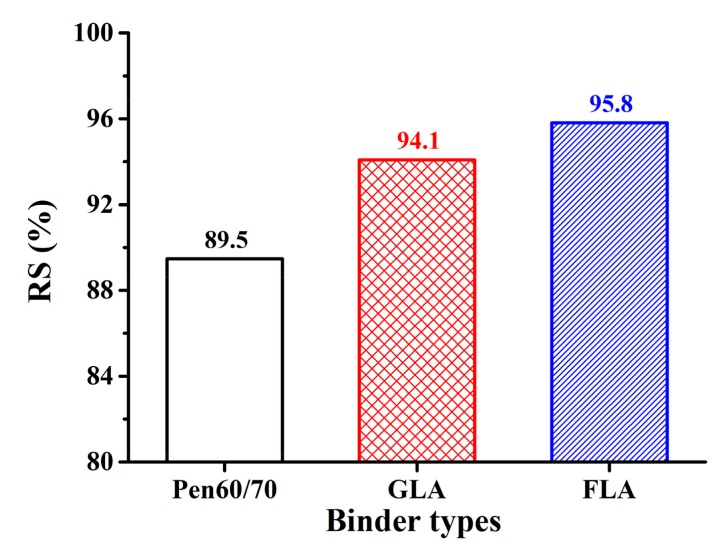
The residual Marshall stability (RS) of test samples.

The Marshall stability and flow value are empirical indexes which quantify the permanent deformation potential of bituminous mixture. Marshall stability evaluates the maximum force the mixture can sustain before failure, while flow values is an index to evaluate the resistance of bituminous mixture to plastic deformation. Figure 11 and Figure 12 and Table 7 illustrate the Marshall properties of specimens before and after 48 h soaking in 60 °C hot water. According to Figure 11a, mixtures with LMA binders exhibited 12–20% higher Marshall stability compared to Pen60/70 mixture. The flow values of LMA mixtures were generally lower than Pen60/70, with the only exception of GLA after 48 h hot water soaking (Figure 11b). The higher Marshall stability and lower flow value of LMA mixture indicated better performance in high service temperature. Residual Marshall stability (RS) is the Marshall stability ratio of sample after 48 h hot water soaking and the original sample. Higher RS value refers to better moisture stability. According to Figure 12, the RS values of GLA and FLA mixtures are 94.1% and 95.8% respectively, while that of Pen60/70 mixture was only 89.5%. The residual Marshall stability results have shown that LMA mixtures have superior resistance to water damage compared to Pen60/70 mixture. 

#### 3.3.2. Indirect Tensile Strength and Moisture Susceptibility

**Figure 13:**
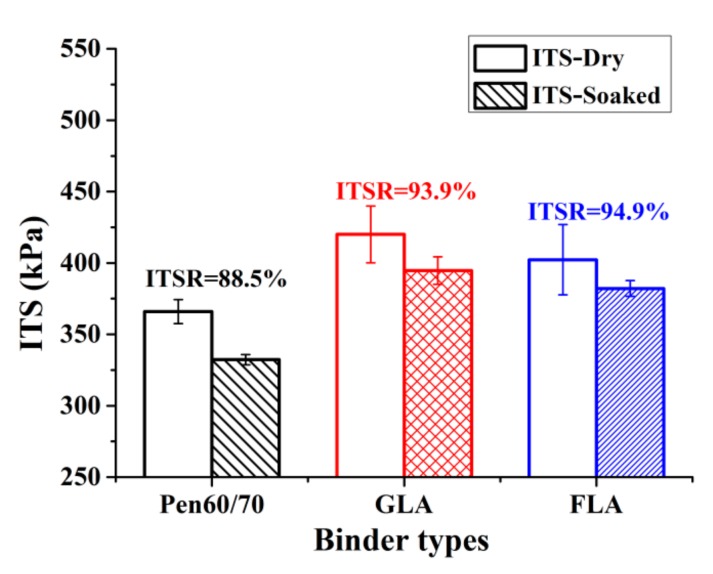
Indirect tensile strength (ITS) test results before and after freeze–thaw cycle.

The residual Marshall stability evaluates the moisture susceptibility in hot water, while TSR (the indirect tensile strength ratio of dry and soaked specimens after one freeze–thaw cycle) characterizes the moisture susceptibility in cold conditions. Figure 13 presents the ITS values of test mixtures before and after freeze–thaw conditioning. It is observed that GLA mixture had the highest ITS in both conditions, followed by FLA and Pen60/70 mixtures. The higher ITS of LMA mixtures are attributed to the stiffness enhancing effect of lignocellulosic modifiers. According to Figure 13, the TSR results of prepared mixtures were in the range between 88% and 95%. All specimens had higher TSR than the minimum required values in specifications, which are always between 70% and 80%. In addition, the TSR values of LMA mixtures were 6–7% higher compared to that of Pen60/70 mixture, showing higher moisture damage resistance in freezing condition (Table 8).

#### 3.3.3. Stiffness Modulus and Aging Resistance

**Figure 14:**
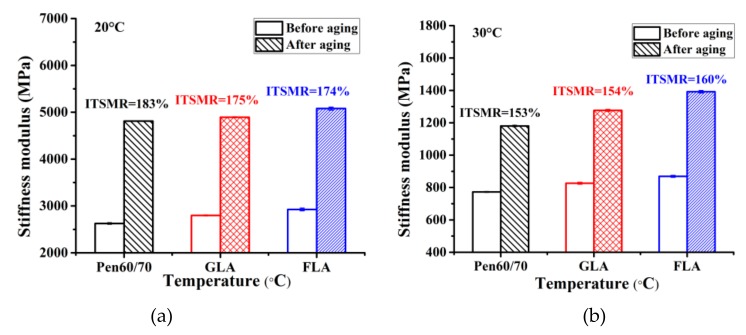
Indirect tensile stiffness modulus (ITSM) test results before and after aging: (**a**) 20 °C and (**b**) 30 °C.

The ITSM results before and after aging condition are presented in Figure 14. In the original state, the ranking of ITSM value from high to low was FLA, GLA and Pen60/70 mixture, at both 20 and 30 °C. Regarding the results after lab-simulated aging, the ranking of ITSM values among different mixtures were identical to that before aging. ITSMR refers to the ratio of the ITSM after and before long-term aging. By comparing the aging index ITSMR, at 30 °C, Pen60/70 mixture exhibited relatively superior aging resistance than LMA mixtures, but at 20 °C the LMA mixtures outperformed Pen60/70 mixture. It is worth noting that the ITSMR values among different mixtures were quite close to each other. Besides, it is observed that the ITSMR values deceased with rising temperature, showing that the bituminous mixture is more sensitive to aging in cooler climate. Finally, it is believed that the LMA mixtures provided similar aging resistance to conventional mixture with Pen60/70 binder. 

## 4. Conclusions

This study comprehensively characterized the feasibly of applying lignocellulosic biomass as a performance enhancer for both bituminous binder and mixture. A series of rheological, mechanical and chemical tests were performed on modified bituminous materials modified with lignocellulosic products. Based on the test results, the following findings can be obtained:

(1) Interaction between lignocellulosic biomass and bituminous binder can be observed.
(2) Lignocellulosic biomass has a slightly negative effect on the workability of bitumen. But lignocellulosic biomass modified binders still meet the viscosity requirements in Superpave specification at 135 °C.
(3) The incorporation of lignocellulosic biomass helps improve the overall service performance of bituminous binder in high, intermediate and low temperatures.
(4) Bituminous mixtures with lignocellulosic biomass modifier binder have superior resistance to rutting and moisture damage. The effect of lignocellulosic biomass on aging resistance is not significant.

Finally, this study brings a better understanding of the potential benefit of lignocellulosic biomass as a performance modifier for bituminous materials. Future study will be conducted on field validation and life cycle assessment of bituminous pavement with different lignocellulosic biomass modifiers.

## Figures and Tables

**Table 1:** Properties of GL and FL.

Test Items	Unit	Value
Diameter	mm	Less than 6
Ash Content (by weight)	%	13–23
Heat Resistance	°C	280 (short time)
Ph Value	-	6.0–8.0
Oil Absorption Rate	%	More than 500
Water Content Rate (by Weight)	%	Less than 5

**Table 2:** SMA 10 gradation used in this research.

BS Sieve Size	Design Data (%)	Passing Requirement (%)
14 mm	100	100
10 mm	96	92–100
5 mm	35	28–42
2.36 mm	26	19–33
75 μm	9.8	7.8–11.8 (including 2% hydrated lime)

**Table 3:** Multiple stress creep recovery (MSCR) test results.

Binder Types	*J*_nr_			% Recovery	
	0.1 kPa (kPa^−1^)	3.2 kPa (kPa^−1^)	*J*_nr_% Diff	0.1 kPa (kPa^−1^)	3.2 kPa (kPa^−1^)
Pen60/70	4.5135	5.0070	10.95	0.70	-0.45
GLA	1.7085	2.2785	34.50	9.65	1.80
FLA	0.5375	1.2340	129.50	30.50	10.70

**Table 4:** Bending beam rheometer (BBR) test result.

Binder Types	−6 °C		−12 °C		−18 °C	
	Stiffness (MPa)	*m*-value (× 10^−2^)	Stiffness (MPa)	*m*-value (× 10^−2^)	Stiffness (MPa)	*m*-value (× 10^−2^)
Pen60/70	175	32.7	280	28.0	541	19.9
GLA	83	37.5	153	31.0	306	26.5
FLA	76	40.2	126	32.8	283	29.2

**Table 5:** Model parameters of sigmoidal function and the Williams–Landel–Ferry (WLF) formula.

Parameters		Pen60/70	GLFMA	FLFMA
**Sigmoidal Function**	δ (Pa)	−0.55919	10.20935	20.22812
	Α (Pa)	8.71694	43,589.56262	58,492.23025
	B (-)	0.25486	0.31567	0.80050
	γ (-)	−0.45159	−0.15908	−0.15820
	R^2^@|G*|	0.99942	0.99696	0.99743
**WLF Formula**	C_1_ (-)	−8.82557	−17.75624	−17.37443
	C_2_ (-)	138.09993	2.36250	2.07013

**Table 6:** Molecular weight distribution.

Sample ID	*M*_p_ (g/mol)	*M*_n_ (g/mol)	*M*_w_ (g/mol)	*M*_z_ (g/mol)	Đ (-)
Pen60/70	917 ± 9	682 ± 27	2371 ± 575	9105 ± 3476	3.4619 ± 0.7065
GLA	672 ± 175	606 ± 17	1917 ± 81	6049 ± 507	3.1619 ± 0.0456
FLA	674 ± 162	565 ± 1	1864 ± 30	6040 ± 107	3.3020 ± 0.0485

**Table 7:** The test results of Marshall stability and flow value test.

Binder Types	Strength (kPa)			Flow Values (mm)	
	Soak (30 min)	Soak (48 h)	RS (%)	Soak (30 min)	Soak (48 h)
Pen60/70	5.7 ± 0.4	5.1 ± 0.5	89.5	4.26 ± 0.9	3.14 ± 0.2
GLA	6.4 ± 0.6	6.0 ± 0.6	94.1	2.90 ± 0.7	3.70 ± 0.1
FLA	6.4 ± 0.3	6.2 ± 0.5	95.8	2.62 ± 0.3	2.70 ± 0.6

**Table 8:** The ITS and ITSR values of test mixtures.

Binder Types	Dry Samples (kPa)	Freeze Samples (kPa)	ITSR (%)
Pen60/70	366 ± 8.4	332 ± 3.6	88.5
GLA	420 ± 19.9	395 ± 8.4	93.9
FLA	402 ± 24.5	382 ± 5.6	94.9

## References

[1] Hendriks A.T.W.M., Zeeman G. (2009). Pretreatments to enhance the digestibility of lignocellulosic biomass. Bioresour. Technol..

[2] Ciolacu D., Ciolacu F., Popa V.I. (2011). Amorphous cellulose-structure and characterization. Cell Chem. Technol..

[3] Pérez J., Muñoz-Dorado J., de la Rubia T., Martínez J. (2002). Biodegradation and biological treatments of cellulose, hemicellulose and lignin: an overview. Int. Microbiol..

[4] Rinaldi R., Jastrzebski R., Clough M.T., Ralph J., Kennema M., Bruijnincx P.C.A., Weckhuysen B.M. (2016). Paving the way for lignin valorisation: Recent advances in bioengineering, biorefining and catalysis. Angew. Chem. Int. Ed..

[5] Mishra P.K., Wimmer R. (2017). Aerosol assisted self-assembly as a route to synthesize solid and hollow spherical lignin colloids and its utilization in layer by layer deposition. Ultrason. Sonochem..

[6] Pawan K.M., Adam E. (2019). The self-assembly of lignin and its application in nanoparticle synthesis: A short review. Nanomaterials.

[7] Karade S.R. (2010). Cement-bonded composites from lignocellulosic wastes. Constr. Build. Mater..

[8] Yang Z., Zhang X., Zhang Z., Zou B., Zhu Z., Lu G., Xu W., Yu J., Yu H. (2018). Effect of aging on chemical and rheological properties of bitumen. Polymers.

[9] Han M., Zeng X., Muhammad Y., Li J., Yang J., Yang S., Wei Y., Meng F. (2019). Preparation of octadecyl amine grafted over waste rubber powder (ODA-WRP) and properties of its incorporation in SBS-modified asphalt. Polymers.

[10] Li J., Han M., Muhammad Y., Liu Y., Su Z., Yang J., Yang S., Duan S. (2018). Preparation and properties of sbs-g-gos-modified asphalt based on a thiol-ene click reaction in a bituminous environment. Polymers.

[11] Yu H., Leng Z., Zhou Z., Shih K., Xiao F., Gao Z. (2017). Optimization of preparation procedure of liquid warm mix additive modified asphalt rubber. J. Cleaner Prod..

[12] Ma T., Wang H., He L., Zhao Y., Huang X., Chen J. (2017). Property characterization of asphalt and mixtures modified by different crumb rubbers. J. Mater. Civ. Eng..

[13] Yu H., Leng Z., Dong Z., Tan Z., Guo F., Yan J. (2018). Workability and mechanical property characterization of asphalt rubber mixtures modified with various warm mix asphalt additives. Constr. Build. Mater..

[14] Yu J., Ren Z., Gao Z., Wu Q., Zhu Z., Yu H. (2019). Recycled heavy bio oil as performance enhancer for rubberized bituminous binders. Polymers.

[15] Yu H., Zhu Z., Zhang Z., Yu J., Oeser M., Wang D. (2019). Recycling waste packaging tape into bituminous mixtures towards enhanced mechanical properties and environmental benefits. J. Cleaner Prod..

[16] Chen H., Xu Q. (2010). Experimental study of fibers in stabilizing and reinforcing asphalt binder. Fuel.

[17] Xu G., Wang H., Zhu H. (2017). Rheological properties and anti-aging performance of asphalt binder modified with wood lignin. Constr. Build. Mater..

[18] Watkins D., Nuruddin M., Hosur M., Tcherbi-Narteh A., Jeelani S. (2015). Extraction and characterization of lignin from different biomass resources. J. Mater. Res. Technol..

[19] Abiola O.S., Kupolati W.K., Sadiku E.R., Ndambuki J.M. (2014). Utilisation of natural fibre as modifier in bituminous mixes: A review. Constr. Build. Mater..

[20] Banerjee P.K., Ghosh M. (2008). Studies on jute–asphalt composites. J. Appl. Polym. Sci..

[21] Chen H., Xu Q., Chen S., Zhang Z. (2009). Evaluation and design of fiber-reinforced asphalt mixtures. Mater. Des..

[22] McCready N.S., Williams R.C. (2008). Utilization of biofuel coproducts as performance enhancers in asphalt binder. Transp. Res. Rec..

[23] Pan T. (2012). A first-principles based chemophysical environment for studying lignins as an asphalt antioxidant. Constr. Build. Mater..

[24] Batista K.B., Padilha R.P.L., Castro T.O., Silva C.F.S.C., Araújo M.F.A.S., Leite L.F.M., Pasa V.M.D., Lins V.F.C. (2018). High-temperature, low-temperature and weathering aging performance of lignin modified asphalt binders. Ind. Crops Prod..

[25] Arafat S., Kumar N., Wasiuddin N.M., Owhe E.O., Lynam J.G. (2019). Sustainable lignin to enhance asphalt binder oxidative aging properties and mix properties. J. Cleaner Prod..

[26] Xie S., Li Q., Karki P., Zhou F., Yuan J.S. (2017). Lignin as renewable and superior asphalt binder modifier. ACS Sustain. Chem. Eng..

[27] Home page of Changzhou Lubisi New Material Technology. www.lbsxcl.com/.

[28] American Society for Testing and Materials (2015). Standard test method for Marshall stability and flow of asphalt mixtures. ASTM D6927–15.

[29] American Association of State and Highway Transportation Officials (2007). Standard method of test for resistance of compacted asphalt mixtures to moisture-induced damage. AASHTO Standard T. 283–07.

[30] American Association of State and Highway Transportation Officials (2006). Penetration of Bituminous Materials. AASHTO T 49.

[31] American Association of State and Highway Transportation Officials (2010). Standard Test Method for Softening Point of Bitumen (Ring-and-Ball Apparatus). AASHTO D 36.

[32] American Association of State and Highway Transportation Officials (2010). Viscosity Determination of Asphalt Binder Using Rotational Viscometer. AASHTO T 316–13.

[33] American Association of State and Highway Transportation Officials (2012). Determining the flexural creep stiffness of asphalt binder using the bending beam rheometer (BBR). AASHTO T 313.

[34] American Association of State and Highway Transportation Officials (2007). Standard Specification for Performance-Graded Asphalt Binder. AASHTO Standard M320.

[35] American Association of State and Highway Transportation Officials (2009). Standard method of test for multiple stress creep recovery (MSCR) test of asphalt binder using a dynamic shear rheometer (DSR). AASHTO TP 70–13.

[36] American Association of State and Highway Transportation Officials (2010). Standard method of test for estimating fatigue resistance of asphalt binders using the linear amplitude sweep. AASHTO TP101.

[37] British Standards Institution (2004). BS EN 12697-26-Bituminous mixtures—Test methods for hot mix asphalt—Part 26: Stiffness.

[38] Yu J., Yu X., Gao Z., Guo F., Wang D., Yu H. (2018). Fatigue resistance characterization of warm asphalt rubber by multiple approaches. Appl. Sci..

[39] Zhou F., Mogawer W., Li H., Andriescu A., Copeland A. (2013). Evaluation of fatigue tests for characterizing asphalt binders. J. Mater. Civ. Eng..

[40] Tang N., Lv Q., Huang W., Lin P., Yan C. (2019). Chemical and rheological evaluation of aging characteristics of terminal blend rubberized asphalt binder. Constr. Build. Mater..

